# Highlights from Heart Rhythm 2018: Updates in Ventricular Tachycardia

**DOI:** 10.19102/icrm.2018.090908

**Published:** 2018-09-15

**Authors:** Jason S. Bradfield

**Affiliations:** ^1^UCLA Cardiac Arrhythmia Center, Ronald Reagan UCLA Medical Center, Los Angeles, CA, USA

**Keywords:** Cardiac anatomy, left ventricular summit, ventricular tachycardia

This year, the majority of the attention at the Heart Rhythm Society Scientific Sessions in Boston, MA was on the results of the Catheter Ablation Versus Antiarrhythmic Drug Therapy for Atrial Fibrillation (CABANA) trial and atrial fibrillation ablation. However, there were also numerous interesting and informative sessions on ventricular tachycardia (VT) ablation.

A clinically essential session was held on mapping and ablation of ventricular arrhythmias originating from the left ventricular (LV) summit, featuring lectures by Drs. Marmar Vaseghi, Wendy Tzou, Andre d’Avila, Fermin Garcia, Miguel Valderrabano, and Roderick Tung^1–6^ and chaired by Drs. Henry H. Hsia and Jason T. Jacobson. The presentations were comprehensive, ranging from talks on underlying anatomy to extensive discussion on epicardial/endocardial/coronary venous/surgical approaches and mapping and ablation.

Historically, premature ventricular complexes and VT were thought to have distinct sites of origin. Ventricular arrhythmias from the anterior mitral annulus, left coronary cusp, aortomitral continuity, great cardiac vein/anterior interventricular vein, and epicardial anterior wall were believed to be distinct entities and in fact have largely been studied/reported as distinct entities in the medical literature. The specific electrocardiogram characteristics and ablation success rates have been reported by numerous investigators over the years. To some extent, this has been helpful and allows physicians to plan ablation procedures and focus on where the highest yield site of mapping is.

Yet, as the electrophysiology community has begun to refocus on understanding patient anatomy, it has become clear that the LV summit is a complex three-dimensional space and that ventricular arrhythmias can exit from any of the above sites, while still having their origin in the LV summit. For instance, the idea of the aortomitral continuity as a site of origin of premature ventricular complexes/VT has never made complete anatomic sense, since this site is composed of fibrous tissue. It is possible that a number of the existing previously reported cases were actually originating from the LV summit and that the earliest site of exit was at the aortomitral continuity.

Given the continuity of these structures, one must clearly understand the anatomy of the region and the relationship with the potential target sites. Further, one must realize that many of these arrhythmias originate deep in the mid-myocardium and that successful ablation may either require the utilization of creative techniques (eg, surgical access, wire mapping/coil embolization, alcohol septal ablation, needle ablation) or a willingness to target the arrhythmia from multiple sites to “triangulate” the deep/distant site of origin (eg, using ablation from the left coronary cusp, below the left cusp, and in the coronary venous system).

Sessions such as this one are what Heart Rhythm is all about: bringing experts together to discuss new and innovative ways to target complex arrhythmias that represent an incredible challenge for practicing electrophysiologists.
